# An uptake and elimination kinetics approach to assess the bioavailability of chromium, copper, and arsenic to earthworms (*Eisenia andrei*) in contaminated field soils

**DOI:** 10.1007/s11356-019-04908-6

**Published:** 2019-03-28

**Authors:** Johanna Kilpi-Koski, Olli-Pekka Penttinen, Ari O. Väisänen, Cornelis A. M. van Gestel

**Affiliations:** 1 Ecosystems and Environment Research Programme, Faculty of Biological and Environmental Sciences, University of Helsinki, Niemenkatu 73, 15140 Lahti, Finland; 2 Department of Chemistry, Jyväskylän yliopisto, PL 35, 40014 Jyväskylä, Finland; 3 Department of Ecological Science, Faculty of Science, Vrije Universiteit, De Boelelaan 1085, 1081 HV Amsterdam, The Netherlands

**Keywords:** Bioavailability, Bioaccumulation, Uptake and elimination kinetics, Metals and metalloids, *Eisenia andrei*, CCA-contaminated soil

## Abstract

The aim of this study was to determine the bioavailability of metals in field soils contaminated with chromated copper arsenate (CCA) mixtures. The uptake and elimination kinetics of chromium, copper, and arsenic were assessed in the earthworm *Eisenia andrei* exposed to soils from a gradient of CCA wood preservative contamination near Hartola, Finland. In soils contaminated with 1480–1590 mg Cr/kg dry soil, 642–791 mg Cu/kg dry soil, and 850–2810 mg Ag/kg dry soil, uptake and elimination kinetics patterns were similar for Cr and Cu. Both metals were rapidly taken up and rapidly excreted by *Eisenia andrei* with equilibrium reached within 1 day. The metalloid As, however, showed very slow uptake and elimination in the earthworms and body concentrations did not reach equilibrium within 21 days. Bioaccumulation factors (BAF) were low for Cu and Cr (< 0.1), but high for As at 0.54–1.8. The potential risk of CCA exposure for the terrestrial ecosystem therefore is mainly due to As.

**Electronic supplementary material:**

The online version of this article (10.1007/s11356-019-04908-6) contains supplementary material, which is available to authorized users.

## Introduction

According to a report from the European Soil Data Centre of the European Commission, the estimated number of potentially contaminated sites in Europe is over 2.5 million. Metals (37.3%) and mineral oil (33.7%) are the most frequent soil contaminants across Europe (van Liedekerke et al. 2014). In Finland, there are around 24,000 contaminated sites, which include 880 sites used for wood salt impregnation and saw mills (Pyy et al. 2013). Chromated copper arsenate (CCA) was used for more than 60 years in Europe and the USA to preserve wooden structures from moss growth and insect damage (Leduc et al. 2008).

In Finland, the use of metal oxides in wood preservatives began in 1950 with the application of Lahontuho K33 (Viitasaari^1991^). In Sweden, they were known as Boliden K33, which became the most widely used CCA formulation. K33 was marketed by many companies around the world under various trade names (Richardson 1993). The CCA compounds are divided into A, B, and C type compounds according to the amount of arsenic, but they also differ in solubility (Viitasaari 1991). Until the end of 1982, the CCA wood preservatives used in Finland were of the type B compound. After that, type C compounds were introduced. Until 2003, CCA wood preservatives were the most popular in the wood impregnation worldwide. At our study site in Finland, wood logs were preserved with K-33.

CCA wood preservatives are effective because of the toxicity of copper and arsenic to fungi and insects (Lebow 1996). However, they have been shown to accumulate in the environment under or near CCA-treated wood (Stilwell and Gorny 1997). Leaching of CCA preservatives into the environment depends on weather conditions and soil characteristics (Balasoiu et al. 2001; Stilwell and Gorny 1997). Reduction of Cr(VI) to Cr(III) is the main driver of a series of reactions in the fixation of CCA complexes, resulting in the insolubilization of CCA. Fixation reactions reduce the leachability of Cu, Cr, and As into the environment but environmental factors like pH and temperature may also affect leachability of these metals (Hingston et al. 2001). The leached CCA metals are expected to adsorb quickly to soil particle surfaces, but may be desorbed into the soil solution after rainfall or irrigation events (Leduc et al. 2008). CCA leaching generally increases with the age of CCA handled timber (Katz and Salem 2005). As a consequence, many terrestrial and aquatic ecosystems are contaminated with leachates of CCA-treated wood.

Little is known about the bioavailability of CCA metals in mixtures, and therefore great uncertainty exists about their potential risk in soils. Measures of bioavailability can be used as a guideline for the risk assessment of soil contamination (Peijnenburg et al. 1997, 1999). Bioavailability of metals is a complicated issue as it depends on the metal itself, the exposed biological species and its ability to regulate metal uptake and excretion, and the environmental compartment where the organism lives (Peijnenburg and Jager 2003). Additionally, the organism’s size, receptor(s), specific pathophysiological characteristics, the metal’s route of entry, the duration and frequency of exposure, the dose and the exposure matrix may also impact bioavailability (Allen et al. 2002). Soil properties like pH (van Gestel and Hensbergen 1997), redox potential (Masscheleyn et al. 1991), clay content (Lin and Puls 2000), Ca concentration, and organic matter content may affect the bioavailability of metals, their kinetics of uptake and elimination in organisms, and the development of body concentrations with time (Vijver et al. 2003*).* Since the toxicity of metals depends on the concentration in the body, uptake and elimination kinetics are important and relevant tools for evaluating the bioavailability of metals.

Long-term contaminated sites may contain many contaminants forming complex mixtures. Metal mixture contamination is different from single metal contamination as different metals may have different kinetics, which will lead to differences in metal concentration ratios in the body compared with external concentrations in exposed organisms. In the mixtures, metal concentration ratios in the body are crucial for determining and understanding toxicity.

The aim of this study was to determinate the bioavailability of chromium, copper, and arsenic to earthworms along a concentration gradient to provide a basis for the ecotoxicological risk assessment of CCA-contaminated field soils. Earthworms are a suitable organism for uptake-elimination kinetic experiments because they have direct contact with the soil and its different compartments. They are also important organisms for soil ecology and its systems (Peijnenburg et al. 1997). We used OECD guideline 317 (OECD 2010) for determining metal uptake and elimination kinetics in earthworms. Our hypotheses were that the uptake kinetics in *Eisenia andrei* are different for Cr, Cu, and As, and do provide insight into the bioavailability of these metals in CCA-contaminated soils.

## Materials and methods

### Study site

The study area was an old wood impregnation site located in Hartola, Southern Finland. In an area measuring 100 m × 150 m, K-33 liquid diluted with water was sprayed with pressure into two impregnation tubes of 12 m × 30 cm and one of 16 m × 3.1 m. At the site, wood logs were preserved with K-33, which contained 34.0% As(V)Oxide, 26.6% Cr_2_O_3_, 14.8% CuO, and 24.6% water. During the period 1958–1966, approximately 2500 wood logs were preserved per year, using 8400 L K-33 liquid annually. After treatment, the wood logs were dried in the area for 3 days. When finishing impregnation actions in autumn, leftover wood impregnation liquid was discarded by pouring onto the soil, leading to contamination of the soil and ground water. At the study site, the pseudo total concentrations of CCA metals in the soil (mg/kg) were as follows: Cr 12.5–1592, As 10.1–2812, and Cu 5.1–79. In Finland, the background levels and the range (mg/kg) for Chromium, Arsenic, and Copper are 31 (6–170), 1 (0.1–25), and 22 (5–110), respectively (Finnish Government decree 214/2007). A detailed description of the Hartola study site has been given by Karjalainen et al. (2009).

### Soil sampling

Humus soil for the experiment was collected from a 60-year-old Norway spruce (*Picea abies* L.) stand of *Oxalis*-*Myrtillus* site type (Cajander 1949) from Hartola, Southern Finland (68°17′820 N/34°44′030 E). The study site (100 m × 150 m) was divided into four sampling areas based on a concentration gradient, being classified as high (H), medium (M), low (L), and control (C) areas (Figure S1 in the Supporting Information). Pre-concentration analysis for Cr, Cu, and As was done on site with a field-portable X-ray fluorescence meter (XRF) (Karjalainen et al. 2009). In each sampling area, five squares (1.5 m × 1.5 m) were laid out from each of which two samples, each of 1 kg, were collected and pooled together. The samples were taken from the humus layer. The depth of the humus layer was 2.5 cm in the highly polluted area, 3.8 cm for medium, 4.1 cm for low, and 5.1 cm in the control area.

### Soil analyses

Soil water holding capacity (WHC) was determined following ISO (1999). Organic matter content (OM) was determined as loss on ignition at 550 °C for 4 h. Soil pH was measured from H_2_O and 0.01 M CaCl_2_ extracts (soil to liquid ratio, 1:10; shaken for 2 h at 200 rpm) with a SCHOTT pH meter, type CG842. Soil moisture content was determined by drying soil samples at 105 °C for 24 h. Particle size distribution was determined using laser size grain analysis, which is based on the forward scattering of monochromatic coherent light as described by Konert and Vandenberghe (1997).

### Uptake/elimination experiment

For the metal uptake and elimination experiment, earthworms (*Eisenia andrei*) were exposed to contaminated soils from the four test sites: high (H), medium (M), low (L), and control (C). Each soil had six replicates per sampling time. One earthworm was placed in a glass container containing 35–50 g of test soil (ww). The earthworms were taken from a synchronized culture at the Vrije Universiteit, Amsterdam, The Netherlands. Only adults with a well-developed clitellum were used. Before starting exposures, the earthworms were acclimatized in OECD artificial soil (OECD 1984) for 24 h at 20 ± 1 °C. OECD artificial soil was used as a control. A small amount of horse dung (2% of the dry soil mass) was mixed in with the test soils for food for the earthworms. Soil moisture content was adjusted to 50% of the WHC. The test containers were loosely covered with a lid, and incubated in climate chambers at 20 ± 1 °C with a light:dark cycle of 16/8 h.

In the uptake phase, six replicate earthworms were sampled at days 0, 0.5, 1, 4, 8, 15, and 21. After 21 days, the remaining earthworms were taken from their respective soils, rinsed with water, and transferred to OECD artificial soil for the elimination phase. Similarly, during the elimination period, six replicate earthworms were sampled at days 0.5, 1, 2, 4, 8, 15, and 21. Sampled earthworms were rinsed with water to remove adhering soil particles, placed on moist filter paper to void their gut for 24 h, weighed, frozen, and freeze-dried for metal analysis.

### Metal analysis

#### Pseudo total metal concentrations in the soils

About 500 mg (dw) soil was weighed into 50-mL plastic bottles and 10 mL aqua regia (HCl:HNO_3_, 3:1) was added. The acids (HCl 36.5–38.0% and HNO_3_ 69.0–70.0%) were supplied by J.T. Baker for trace metal analysis. After closing, the bottles were placed in an ultrasonic bath (Transsonic 820/H Elma®) for 3 × 3 min at a temperature of about 45–50 °C. After sonification and cooling, the samples were filtered (Whatman no. 41) into a 25-mL volumetric glass bottle, diluted with high purity ELGA water to a volume of 25 mL, and stored in plastic bottles for the analysis with Inductively Coupled Plasma-Optical Emission Spectrometry (ICP-OES; Perkin-Elmer Optima 4300DV). All equipment was rinsed with acid before use. Reference materials for contaminated soils, SRM 2710 and SRM 2711, both certified by the National Institute of Standards and technology (NIST), were included in the analysis. Recoveries from the certified reference sample SRM 2710 were 96% for As and 92% for Cu; recovery of Cu from the reference material SRM 2711 was 96%. No certified reference values were given for Cr concentration in the SRM 2710 sample. The procedure has been described by Väisänen et al. (2002). Detection limits for pseudo total concentrations of Cr, Cu, and As were 0.3, 0.4, and 2 mg/kg dry soil.

#### Extractable metals

To determine available metal concentrations, the test soils were extracted with H_2_O and 0.01 M CaCl_2_. About 5 g moist test soil was extracted with 50 mL H_2_O or 50 mL 0.01 M CaCl_2_ by shaking for 2 h at 200 rpm. After settling overnight, pH was measured, and samples were 0.45-μm-filtered and preserved with HNO_3_ for analyzing extractable metal concentrations (Smit et al. 1997) with ICP-OES. Detection limits for 0.01 M CaCl_2_-extractable concentrations were 0.03, 0.04, and 0.2 mg/kg dry soil and for H_2_O-extractable, 0.08, 0.13, and 0.7 mg/kg dry soil, respectively.

#### Metal concentrations in *Eisenia andrei*

The earthworms were digested individually in 4 mL aqua regia (3 HCl:1 HNO_3_). Earthworm samples were placed for 1 h in a water bath at 70–80 °C. After cooling, the extract was filtered and diluted with high purity ELGA water to a volume of 25 mL (Lukkari et al. 2004). The samples were analyzed for metal concentrations by ICP-OES (Perkin-Elmer (Norwalk, CT, USA) model Optima 4300 DV) as described by Väisänen et al. (2002). A Scott-type double-pass spray chamber and a cross-flow nebulizer were used throughout. The determination of metal concentrations was performed using default parameters of the instrument (nebulizer flow 0.6 L min^−1^, auxiliary gas flow 0.2 L min^−1^, plasma gas flow 15 L min^−1^, and plasma power of 1400 W). The wavelengths with the axial plasma viewing used in the determination were 193.696 nm, 283.563 nm, and 324.752 nm for As, Cr, and Cu, respectively. Quality control was performed by the analysis of two certified reference materials, DOLT-4 Dogfish liver and TORT-2 Lobster hepatopancreas. High recoveries were obtained for all the elements of interest. The certified and measured concentrations (mg/kg) and recoveries of metals (%) from the DOLT-4 Dogfish liver were as follows: As 9.66 ± 0.62, 7.75 ± 0.08, and 80.2; Cu 31.2 ± 1.1, 30.3 ± 0.4, and 97.1; Cr 1.4 (stated value, not certified), 1.4 ± 0.4, and 100. The certified and measured concentrations (mg/kg) and metal recoveries (%) from the TORT-2 Lobster hepatopancreas were as follows: As 21.6 ± 1.8, 22.9 ± 0.3, and 105.8; Cu 106 ± 10, 97 ± 2, and 92, and Cr 0.77 ± 0.15, 1.0 ± 0.2, and 130, respectively.

### Kinetics model

A one-compartment model was applied to describe the uptake (Eq. 1) and elimination (Eq. 2) kinetics of chromium, copper, and arsenic in the earthworms exposed to the Hartola soils (Atkins 1969).

1 $$ {C}_{\mathrm{worm}}={C}_0+\frac{k1}{k2}\times {C}_{\exp 1}\times \left(1-{e}^{-{k}_2t}\right) $$

2 $$ {C}_{\mathrm{worm}}={C}_0+\frac{k1}{k2}\times {C}_{\exp 1}\times \left(1-{e}^{-{k}_2t}\right)+\frac{k_1}{k_2}\times {C}_{\exp 1}\times \left(1-{e}^{-{k}_2\left(t-{t}_x\right)}\right) $$

where *C*_worm_ is the internal copper/chromium/arsenic concentration in the earthworms at time *t* (mg/kg dry body weight), *C*_0_ is the initial (background) copper/chromium/arsenic concentration in the earthworms at *t* = 0 (mg/kg dry body weight), *k*_1_ is the uptake rate constant (kg soil/kg earthworm/day), *k*_2_ is the elimination rate constant (day^−1^), *C*_exp1_ is the copper/chromium/arsenic exposure concentration during the uptake phase (mg/kg dry soil), *t* is the exposure time (days), and *t*_*x*_ is the day on which animals were transferred to clean OECD artificial soil (day 21). Both Eqs. 1 and 2 were fitted together to obtain single values for the uptake and for the elimination rate constants. Microsoft Excel 2010 was used to fit the one-compartment model to the data for each study site and metal, and IBM SPSS Statistics 21 to estimate the standard errors and other statistical parameters of the estimated uptake and elimination rate constants. The bioaccumulation factor (BAF) for the accumulation of the metals in *E. andrei* was estimated using the following equation (Sharma et al. 2011): $BAF=\frac{k_1}{k_2}$

## Results

The CCA-contaminated field soils from Hartola were acidic with pH_CaCl2_ between 3.4 and 4.5 and pH_H2O_ between 4.3 and 5.7 (Table 1). Organic matter contents (OM) were high, ranging from 21 to 32%, and soils were sandy with low clay content. Water holding capacity (WHC) of the study soils was high, with 238–325%. Moisture contents ranged from 41.6 to 93.8%.

**Table 1:** Properties of the CCA-contaminated soils from Hartola, Finland. Shown are mean values with standard deviation; OM organic matter, WHC water holding capacity

Site	pH_CaCl2_ (*n* = 3)	pH_H2O_ (*n* = 3)	%OM (*n* = 3)	WHC (*n* = 10)	Moisture content (%) (*n* = 5)	% clay (< 8 μm) (*n* = 5)	% silt (8–64 μm) (*n* = 5)	% sand (64–2000 μm) (*n* = 5)
Control	3.39 ± 0.05	4.30 ± 0.10	30.8 ± 2.63	325 ± 77	49.6 ± 2.94	5.1 ± 1.6	11.1 ± 1.7	83.7 ± 3.3
Low	3.89 ± 0.01	4.95 ± 0.10	23.9 ± 6.62	271 ± 124	50.5 ± 9.62	4.1 ± 0.8	10.3 ± 0.8	85.6 ± 1.6
Medium	4.32 ± 0.07	5.53 ± 0.02	31.8 ± 4.23	277 ± 56	93.8 ± 11.6	4.8 ± 0.6	12.6 ± 1.6	82.6 ± 1.8
High	4.53 ± 0.08	5.68 ± 0.07	20.8 ± 5.50	238 ± 105	41.6 ± 11.2	3.1 ± 2.7	6.5 ± 1.7	90.4 ± 4.2

Pseudo total concentrations (mg/kg) of chromium and copper in the high and medium contaminated areas were similar, while they were low in the low contaminated and control areas (Table 2). Total As concentration was highest in the high contaminated soil. Water and 0.01 M CaCl_2_ extractable metal concentrations were below the detection limit for the control and low contaminated soils. For Cr and As, water-extractable concentrations in the high and medium contaminated soils were slightly higher than the CaCl_2_ extractable concentrations. For copper, the difference between water- and CaCl_2_-extractable concentrations in these soils was small and not consistent (Tables 2).

**Table 2:** Mean (± SD) pseudo total, water, and 0.01 M CaCl_2_ extractable concentrations [mg/kg dry soil] of chromium (Cr), copper (Cu), and arsenic (As) in the CCA-contaminated soils from Hartola, Finland, used for the toxicokinetics experiment with *Eisenia andrei*

Study site	Pseudo total [mg/kg] (*n* = 37–39)			H_2_O extractable [mg/kg] (*n* = 3)			CaCl_2_ extractable [mg/kg] (*n* = 3)		
	Cr	Cu	As	Cr	Cu	As	Cr	Cu	As
Control (C)	5.34 ± 1.68	4.71 ± 1.06	6.12 ± 1.22	<	0.21 ± 0.11	0.20 ± 0.11	<	<	3.54 ± 0.09
Low (L)	12.5 ± 10.6	5.14 ± 5.3	10.1 ± 5.5	<	0.19 ± 0.04	0.17 ± 0.03	<	<	3.43 ± 0.11
Medium (M)	1590 ± 247	791 ± 140	850 ± 225	4.72 ± 0.38	2.95 ± 0.24	9.98 ± 0.62	0.84 ± 0.16	4.58 ± 0.23	7.67 ± 0.29
High (H)	1480 ± 355	642 ± 180	2810 ± 921	13.5 ± 0.33	6.23 ± 0.13	54.5 ± 1.94	2.55 ± 0.39	2.22 ± 0.26	20.8 ± 2.54

<, below detection limit

**Table 3:** Kinetics parameters (± SE) for the uptake and elimination of Cr, Cu, and As in the earthworm *Eisenia andrei* following exposure to CCA field-contaminated soils from Hartola, Finland. Kinetics parameters were derived by relating metal concentrations in the earthworms to pseudo total concentrations in the test soils. *k*_1_ is the uptake rate constant, *k*_2_ the elimination rate constant, and BAF is bioaccumulation factor. A one-compartment model was used to estimate kinetics parameters, using Eq. 1 for uptake and Eq. 2 for elimination phase data. See Fig. 1 for the corresponding data and model fits

Site	*k*_1_ (kg soil/kg worm/day)			*k*_2_ (day^−1^)			BAF		
	Cr	Cu	As	Cr	Cu	As	Cr	Cu	As
Low	–	–	–	–	–	–	–	–	–
Medium	0.27 ± 0.68	0.19 ± 0.042	0.011 ± 0.0091	7.6 ± 19	2.2 ± 0.52	0.0062 ± 0.0048	0.036	0.086	1.8
High	0.71*	0.16 ± 0.043	0.0065 ± 0.00055	24.6*	2.4 ± 0.65	0.012 ± 0.0052	0.029	0.067	0.54

*Very large SE

Earthworm survival was high during the 42-day experimental period, with only 3 animals dying in the control (83 worms) and 2, 7, and 11 dead worms out of 83 in low, medium, and high contaminated soils, respectively.

**Fig. 1:**
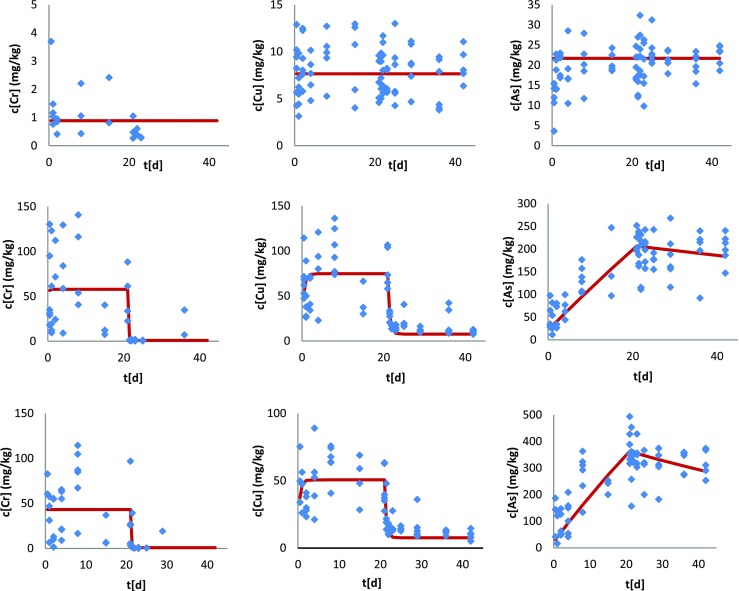
Uptake and elimination kinetics of chromium (left), copper (middle), and arsenic (right) in earthworms (*Eisenia andrei*) exposed for 21 days to low (top), medium (middle), and high (bottom) CCA-contaminated field soils from the site near Hartola, Finland, followed by a 21-day elimination phase in clean OECD artificial soil. Lines show the fit of a one-compartment model to the data (Eqs. 1 and 2). Table 3 shows the corresponding uptake and elimination rate constants.

In the low contaminated soil, no uptake of chromium, copper, and arsenic was seen, with earthworm body concentrations remaining more or less constant at approximately 0.9, 7.7, and 22 mg/kg body weight, respectively throughout the uptake and elimination phases (Fig. 1). In the medium and high contaminated soils, chromium and copper showed very fast uptake and elimination kinetics in the earthworms. Equilibrium was reached within a day, and after transfer of the earthworms to clean soil, Cr and Cu body concentrations returned to the background level also within 1 day. Arsenic, however, showed very slow uptake and elimination kinetics in the earthworms exposed to the medium and high contaminated soil. Steady state was not reached within 21 days of exposure (Fig. 1).

There was little difference between Cr and Cu uptake (*k*_1_) and elimination (*k*_2_) rate constants for medium and high contaminated soils, and they were indicating very fast kinetics. For As, *k*_1_ and *k*_2_ in both the high and medium contaminated soils were very small, indicating slow kinetics. Bioaccumulation factors (BAF) were well below 0.1 for Cr and Cu, but between 0.54 and 1.8 for As, and did not differ much for the medium and high contaminated soils.

Calculating uptake rate constants (*k*_1_) based on water- or CaCl_2_-extractable concentrations (Tables S1 and S2) did slightly decrease the difference between medium and high contaminated soils for Cr but not for Cu and As.

## Discussion

The main objective of this study was determining the bioavailability of chromium (Cr), copper (Cu), and arsenic (As) to the earthworm *E. andrei* along a gradient of metal pollution, applying a toxicokinetics approach. Test soils were taken from a gradient of Cr, Cu, and As contamination in a Finish forest field soil contaminated several decades with a chromated copper arsenate (CCA) wood preservative. In an earlier study, Karjalainen et al. (2009) indicated that pollution levels in this area may be hazardous to the terrestrial ecosystem. In the present study, we found two different uptake-elimination patterns in *E. andrei.* Uptake rate constant *k*_1_ and elimination rate constant *k*_2_ were high indicating fast kinetics for Cu and Cr, but were very low showing very slow kinetics for As. These differences may be caused by differences in the bioavailability of the metals and their metabolic routes in earthworms.

### Metal availability

Variation of bioavailability and accumulation has been studied for different chemicals and earthworm species and using different experimental designs and analytical platforms (Spurgeon et al. 2011). These studies have shown a difference in bioavailability between artificial soils (like OECD) and field soils (Peijnenburg et al. 1999). Metals are more bioavailable in freshly spiked artificial soils than in field-contaminated soils (Spurgeon and Hopkin 1995). Pseudo total concentrations (mg/kg, dw) of Cu, Cr, and As in the medium and high CCA field-contaminated soils (Table 2) were 6–8, 15–16, and 17–56 times higher than the Finnish lower limit values, respectively. Although pseudo total metal concentrations were high, similar to e.g., Hagner et al. (2017), this did not translate into high available concentrations in H_2_O and 0.01 M CaCl_2_ extracts. This was expected because the field soil was contaminated decades ago. The metal contaminant pool requires time to diffuse into micro or nanopores and to be absorbed into organic matter and adsorbed onto soil particles (Allen et al. 2002). This aging process, which usually is nearly completed within 1 year of spiking the metals, makes a direct and straightforward comparison of metal bioavailability in OECD artificial and field soils challenging (Peijnenburg et al. 1999).

In this study, the bioavailability of Cu, Cr, and As metals and their partitioning in the field-contaminated soils were influenced by soil properties like soil pH, organic matter, and clay content. The CCA-contaminated field soils were acidic (pH_CaCl2_ 3.4–4.5), which has influenced speciation and mobility of the metals present. Under acidic conditions, the toxic and mobile Cr(VI) will be reduced to the stable and less toxic Cr(III) (Kumpiene et al. 2008; Sivakumar and Subbhuraam 2005). In soil, copper is bound to organic matter. When pH increases the sorption of the free Cu^2+^, ion on solid organic matter increases (Degryse et al. 2009) and Cu also becomes more strongly bound to oxide surfaces (Khaodhiar et al. 2000). Peijnenburg et al. (1999) did not observe significant uptake of arsenic in earthworms at pH_CaCl2_ < 6 and at pH_CaCl2_ > 6.75. At low pH and high redox potential, As is mainly in the As(V) form, but when pH increases and redox potential decreases, As(III) is the dominant form in soils (Masscheleyn et al. 1991) and in earthworms (Lee and Kim 2013). Soil acidity was found to be the most important solid-phase characteristic modulating the availability of As and its sorption (Balasoiu et al. 2001; Peijnenburg et al. 1999). All these studies support our findings of low H_2_O- and 0.01 M CaCl_2_-extractable concentrations of Cr, Cu, and As in the acidic and high organic CCA-contaminated field soils from Hartola.

Many studies have shown that metals bind to organic matter and, as a consequence, are not available for uptake. Cr, Cu, and As all have high affinity for binding to soil organic matter (Meharg et al. 1998; Peijnenburg et al. 1999; Marinussen et al. 1997; Speir et al. 1995). In this study, organic matter contents (OM) were high, ranging between 21 and 32%, while the Hartola soils were sandy with low clay content. Balasoiu et al. (2001) concluded that Cu was bound to organic matter because of suitable reactive groups and retained by complexation rather than ion exchange. Chromium partitioning to organic matter was similar to that of copper (Balasoiu et al. 2001). In high organic soils, Cr and Cu are present in less mobile and less available forms (Balasoiu et al. 2001; Gupta et al. 1996; Maiz et al. 2000). Also, As has high affinity for binding to soil organic matter (Meharg et al. 1998). These findings may explain the low H_2_O and CaCl_2_ extractable concentrations in our test soils (Table 2). In our test soils, the available concentrations of Cr, Cu, and As were very low in the low contaminated soil, and in general, less than 1% of the metal was available in the medium and high contaminated soils, except for the water extractability of As which was 1–2%. The slightly higher metal availability in the high contaminated soil compared with the medium contaminated soil (Table 2) can be attributed to the higher organic matter and clay content of the latter. The fact that for Cr and As water-extractable concentrations were higher than CaCl_2_-extractable concentrations suggests that these elements were not present as cations in the CCA-contaminated field soils.

### Uptake—elimination kinetics of chromium and copper

In the present study, differences in H_2_O or 0.01 M CaCl_2_ extractability did not translate into differences in uptake kinetics in the earthworms. Cr and Cu accumulated very rapidly in *E. andrei* and steady-state concentrations were reached within 1 day of exposure. Peijnenburg et al. (1999) found similar patterns for Cr and Cu. Internal steady-state concentrations (day 21) in *E. andrei* exposed to the medium and high contaminated soils were 88.2 and 97.1 mg/kg dw, respectively for Cu, and 106 and 63.6 mg/kg dw, respectively for Cr. van Gestel et al. (1993) exposed *E. andrei* for 3 weeks to Cr concentrations of 0, 10, 32, 100, 320, and 1000 mg/kg dry soil in freshly spiked OECD artificial soil and found tissue concentrations in the earthworms of 0.8–18 mg/kg dw. In Dutch field soils, containing total concentrations of 3.2–988 mg Cr/kg dry soil and 1.1–108 mg Cu/kg dry soil, steady-state concentrations in *E. andrei* were 1.04–14.0 mg Cr/kg dw and 5.72–34.4 mg Cu/kg dw (Peijnenburg et al. 1999). Following exposure to field-contaminated soils from the UK, containing total concentrations of 725 and 1732 mg Cu/kg dry soil, tissue concentrations in *Lumbricus rubellus* were 44.1 and 85.3 mg Cu/kg dw (Langdon et al. 2001). The latter findings are similar to the values obtained in the present study, but the levels of Cr in soils and *E. andrei* in the studies of van Gestel et al. (1993) and Peijnenburg et al. (1999) are lower than in the present study. The differences between studies can be explained by differences in pH and organic matter content of the field soils (Langdon et al. 2001; Peijnenburg et al. 1999), and the use of (freshly spiked) artificial soil (van Gestel et al. 1993).

In the medium (M) and high (H) contaminated soils, fast uptake kinetics of chromium and copper with fast elimination rates were seen in the earthworm *E. andrei*. The absence of clear differences between the two soils might be due to the fact that these soils had very similar pseudo total metal concentrations. Uptake rate constants *k*_1_ for Cr in medium and high soils were 0.27 and 0.71 kg soil/kg worm/day, respectively, and for Cu, 0.19 and 0.16 kg soil/kg worm/day, respectively. Equilibrium was reached within a day and after transfer of the earthworms to clean OECD soil, they reached the background level also within 1 day. Nahmani et al. (2009) found similar uptake rate constants *k*_1_ (0.16–0.57 kg soil/kg worm/day) for Cu in different UK field soils. In our study, very fast elimination of Cr and Cu from *E. andrei* occurred with *k*_2_ = 7.6–24.6 and 2.2–2.4 day^−1^, respectively. These *k*_2_ values agree with the data of Peijnenburg et al. (1999), Spurgeon and Hopkin (1999), and van Gestel et al. (1993). Nahmani et al. (2009) found the opposite effect for the elimination of Cu. Compared with their soils, our field soils had higher organic matter contents and were more acidic.

Why are uptake and elimination patterns of Cu and Cr similar? Cr may be mimicking the essential metal Cu. Cr also is an essential nutrient playing a role in the release of insulin from tissues when needed for the usage of sugars, proteins, and fats (Shrivastava et al. 2002). In acidic environments, Cu and Cr occur as cations (Cu^2+^ and Cr^3+^). Their size is similar and they have very similar chemical characteristics. Both are Lewis acids which bind strongly to organic matter forming similar type organic ligands, which may explain why these two metals have similar uptake and elimination patterns in *E. andrei* like other essential metals. Fast uptake reaching equilibrium within a few days for other essential metals like Zn and non-equilibrium for non-essential metals were reported by Spurgeon and Hopkin (1999). The metabolic routes of copper and chromium, however, are different in earthworms. In this study, the fast elimination may indicate that copper is detoxified mainly by excretion, which is supported by Spurgeon and Hopkin (1999). Copper is needed in biochemical reactions as it is part of numerous enzymes (Fisker et al. 2013) transporting substances in cells and tissues but hardly accumulating itself in earthworms (Kennette et al. 2002). Earthworms can regulate copper (Fisker et al. 2011) using metallothionein proteins. Cu itself is not so active in inducing metallothionein-gene expression, but might need an induction of the protein by other agents, like Cd, to facilitate its binding to earthworm MTs. If metallothioneins are induced by Cd, the Cu concentrations are higher in earthworms (Mariño et al. 1998). Chromium(VI) is accumulated inside the cell through the same membrane channels used for the transfer of isoelectric and isostructural anions, like SO_4_^2−^ and HPO_4_^2−^. Glutathione, which is present in high concentrations, plays an important role in the intracellular metabolism of Cr(VI). There are several mechanisms for Cr(VI) reduction to Cr(III) via intermediates like Cr(V) and Cr(IV) (Codd et al. 2001) by Jurket cells (Shi et al. 1999). In the reduction process, superoxide radicals (·O_2_), H_2_O_2_, and hydroxyl (·OH) radicals, collectively called oxygen species (ROS), play a major role as a messenger for NF-kB activation in Jurket cells (Shi et al. 1999). Environmental concern is triggered by Cr(VI), which is more mobile and more toxic than Cr(III) for *Eisenia fetida* (Sivakumar and Subbhuraam 2005). In the present study, a reduction from Cr(VI) to Cr(III) may have happened already in the soils because of their high organic matter contents (Speir et al. 1995).

The bioaccumulation factor (BAF) calculated to estimate metal bioavailability in the different soils was low (< 0.1) for Cr and Cu due to fast excretion from the earthworms. BAFs for Cu in *E. fetida* exposed to different contaminated field soils from UK were higher at 0.18–1.25 (Nahmani et al. 2009). Langdon et al. (2001) determined BAF for Cu accumulation in *L. rubellus* exposed to two different soils in UK of 0.060 and 0.049, which are similar to the values found in the present study. BAF values for Cr accumulation in *E. andrei* were 0.031–0.047 when exposed to Cr in freshly spiked artificial soil (van Gestel et al. 1993). Also, these BAFs are similar to the values measured in the present study.

### Uptake—elimination kinetics of arsenic

In the low (L) contaminated field soil, no uptake of arsenic was seen, with earthworm body concentrations remaining more or less constant at approximately 22 mg/kg body weight, throughout the uptake and elimination phases. This matches with the low availability of As in this soil seen from H_2_O and CaCl_2_ extractions (Table 2). Very slow uptake and elimination kinetics were seen in *E. andrei* upon exposure to the medium (M) and high (H) contaminated Hartola field soils, and steady-state was not reached after 21-day exposure. Similar uptake patterns have been reported in field soil (Peijnenburg et al. 1999) and in artificial soils (Lee and Kim 2013). Langdon et al. (2003a) found that *L. rubellus* eliminated arsenic from their tissues over a 21-day experimental period, which differs from our results. The difference between *L. rubellus* and *E. fetida*/*E. andrei* may be due to fact that *L. rubellus* from contaminated sites have developed tolerant to arsenic toxicity (Langdon et al. 1999) by developing an efficient arsenic elimination mechanism (Langdon et al. 2003a). Fisher and Koszorus (1992) and Peijnenburg et al. (1999) found no As elimination over an 8-week period in *E. fetida*, which agrees with the absence of any As elimination in *E. andrei* in the present study.

Uptake rate constants *k*_1_ values for As were 0.011 and 0.0065 kg soil/kg worm/day, respectively, which agrees with the *k*_1_ of 0.0046 kg soil/kg worm/day reported by Peijnenburg et al. (1999) for *E. andrei* exposed to different Dutch field soils. Also, Lee and Kim (2013) found no equilibrium of As uptake and elimination in *E. fetida* within 28-day exposure. In the present study, elimination rate constants for As were 0.0062 day^−1^ for the medium site and 0.012 day^−1^ for the high site. This slow elimination kinetics is supported by Lee and Kim (2013) and Peijnenburg et al. (1999). As a consequence of the slow elimination, steady-state was not reached within 21 days (Peijnenburg et al. 1999; Lee and Kim 2013), which is in agreement with the results of the present study. González-Alcaraz and van Gestel (2016), however, found that As body concentration of *E. andrei* increased very fast (*k*_1_ = 1.10 kg soil/kg earthworm/day; 20 °C and 50%WHC), reaching steady-state after 1–3 days of exposure of contaminated soils from a mine tailing. The elimination rate constant *k*_2_ = 12.7 day^−1^ (20 °C and 50%WHC) was much higher than in the present study. This fast kinetics of As is totally opposite to our findings, and is probably due to the different soil types, especially the high pH_CaCl2_ (6.04–7.44) and low organic matter contents (~ 1.5–4.3%) of the soils used in the study of González-Alcaraz and van Gestel (2016).

Earthworms may take up arsenic mainly via the alimentary route (Morgan et al. 1994; Langdon et al. 1999). In earthworm tissues, As is sequestered as As-thiol complexes (Morgan et al. 1994) while also other metal-chelating proteins, metallothioneins (MTs), may be involved in As binding (Langdon et al. 2005) causing its bioconcentration in earthworms (Lee and Kim 2013). Slow excretion of As from *E. andrei* indicates sequestration in less-toxic forms without elimination (Meharg et al. 1998). Fisher and Koszorus (1992) concluded that As may have restricted ability for elimination which also agrees with our results and may explain why As accumulated to *E. andrei* in the present study.

The BAFs for As of 1.8 and 0.54 for the medium and high sites, respectively, show that As is bioaccumulated by *E. andrei*. Langdon et al. (2001) calculated a very low BAF, suggesting that different earthworm species detoxified As in different ways. Their field soils differed from this study in having low organic matter contents (1.58–10.02%) and pH(H_2_O) 4.71–7.18, while the history of As contamination in the mining area has been very long and As speciation form probably was different from our study. In the mining area, arsenic is accumulated as an arsenobetaine form (Langdon et al. 2005) which is produced by the earthworms upon metabolism of arsenate (Langdon et al. 2003b). The BAF of 0.64 reported by Peijnenburg et al. (1999) for *E. andrei* exposed to Dutch field soils based on steady state concentrations is similar to our data, suggesting As speciation was similar to our soils.

## Conclusion

Little is known about the bioavailability of the metals in the CCA mixture, leading to great uncertainty about the potential risk of CCA-contaminated soils. In this study, we used a toxicokinetics approach to assess the bioavailability of copper, chromium, and arsenic to earthworms (*Eisenia andrei*) in CCA-contaminated field soils. All three metals were available at the site, but the uptake and elimination patterns in *E. andrei* of Cr, Cu, and As were quite different. Uptake and elimination for the essential metals Cr and Cu were very fast with equilibrium being reached within 1 day, probably due to active regulation of the body concentrations by the earthworms. For As, uptake and elimination kinetics were very slow leading to relatively high bioaccumulation factors (BAF), suggesting potential risk of metal biomagnification in the food chain. When assessing the ecological risk of CCA-contaminated soils in Hartola, Finland, focus should especially be on the high bioavailability of As, and consider its possible transfer in the food chain. Further research is needed to study the consequences of exposure to multiple metals at this site.

## Electronic supplementary material

ESM 1 (DOCX 439 kb)
