# Supplementary material for: An uptake and elimination kinetics approach to assess the bioavailability of chromium, copper, and arsenic to earthworms (Eisenia andrei) in contaminated field soils
Source: Environ Sci Pollut Res Int. 2019 Mar 28;26(15):15095–104. doi: 10.1007/s11356-019-04908-6 (PMC6529395; doi:10.1007/s11356-019-04908-6)
Supplement: Supplementary file 1 — (DOCX 439 kb) [file 11356_2019_4908_MOESM1_ESM.docx]

**Supporting Information to**

“A toxicokinetics approach to assess the bioavailability of chromium, copper and arsenic to earthworms (*Eisenia andrei*) in contaminated field soils”

Johanna Kilpi-Koski*, Olli-Pekka Penttinen*, Ari O. Väisänen^#^ and Cornelis A.M. van Gestel^§^

*Department of Environmental Sciences, Faculty of Biological and Environmental Sciences, University of Helsinki, Niemenkatu 73, 15140 Lahti, Finland

^#^Department of Chemistry, PL 35, 40014 Jyväskylän yliopisto, Jyväskylä, Finland

^§^Department of Ecological Science, Faculty of Science, Vrije Universiteit, De Boelelaan 1085, 1081 HV, Amsterdam, The Netherlands


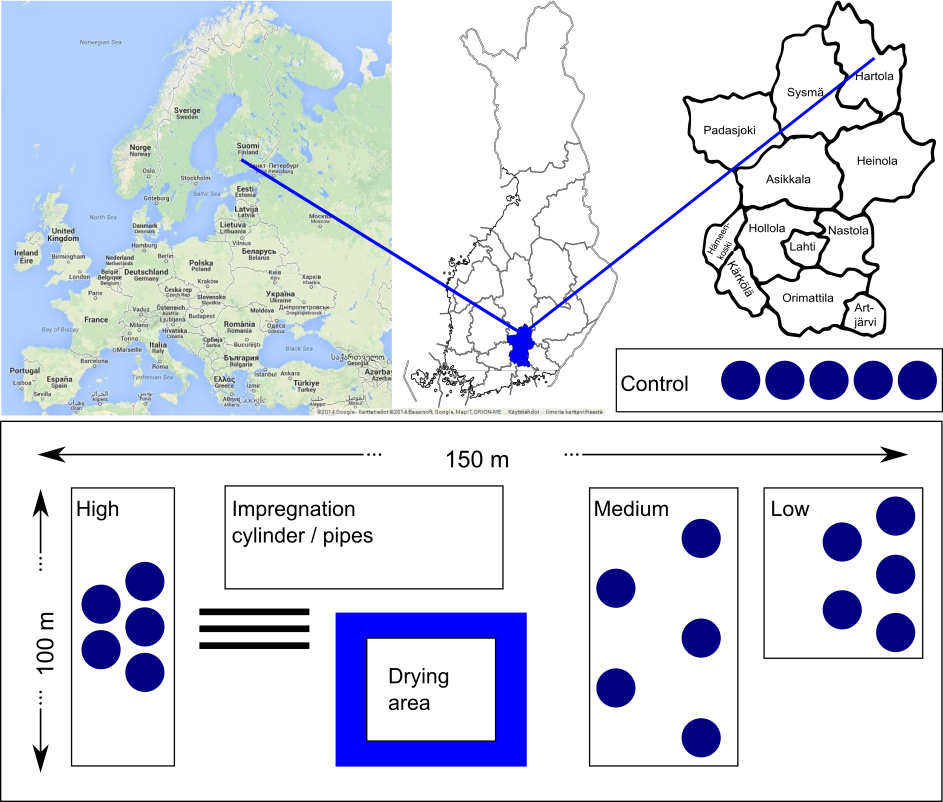


FIGURE S1. Location of the chromated copper arsenate (CCA)-contaminated field site near Hartola, Finland and schematic outline of the locations where soil samples were taken for the bioaccumulation experiments with earthworms.

Table S1: Kinetics parameters (±SE) for the uptake and elimination of Cr, Cu and As in the earthworm *Eisenia andrei* following exposure to CCA-field contaminated soils from Hartola, Finland. Kinetics parameters were derived by relating metal concentrations in the earthworms to H_2_O-extractable concentrations in the test soils. k_1_ is the uptake rate constant and k_2_ the elimination rate constant. A one-compartment model was used to estimate kinetics parameters, using equation 1 for uptake and equation 2 for elimination phase data.

| **Site** | **k_1_ (kg soil/kg worm/day)** | | | **k_2_ (day^-1^)** | | |
| --- | --- | --- | --- | --- | --- | --- |
|  | Cr | Cu | As | Cr | Cu | As |
| **Low** | - | - | - | - | - | - |
| **Medium** | 91.2±228 | 50.5±11.4 | 0.941±0.08 | 7.6± 19 | 2.2± 0.52 | 0.0062± 0.0048 |
| **High** | 77.2 * | 16.4±4.41 | 0.336±0.28 | 24.6 * | 2.4± 0.65 | 0.012± 0.0052 |

*very large SE

Table S2: Kinetics parameters (±SE) for the uptake and elimination of Cr, Cu and As in the earthworm *Eisenia andrei* following exposure to CCA-field contaminated soils from Hartola, Finland. Kinetics parameters were derived by relating metal concentrations in the earthworms to CaCl_2_-extractable concentrations in the test soils. k_1_ is the uptake rate constant and k_2_ the elimination rate constant. A one-compartment model was used to estimate kinetics parameters, using equation 1 for uptake and equation 2 for elimination phase data.

| **Site** | **k_1_ (kg soil/kg worm/day)** | | | **k_2_ (day^-1^)** | | |
| --- | --- | --- | --- | --- | --- | --- |
|  | Cr | Cu | As | Cr | Cu | As |
| **Low** | - | - | - | - | - | - |
| **Medium** | 510±1270 | 32.5±7.33 | 1.22±0.10 | 7.6± 19 | 2.2± 0.52 | 0.0062± 0.0048 |
| **High** | 386* | 46.0±12.4 | 0.881±0.074 | 24.6 * | 2.4± 0.65 | 0.012± 0.0052 |

*very large SE
